# Aging and Parkinson’s Disease: Different Sides of the Same Coin?

**DOI:** 10.1002/mds.27037

**Published:** 2017-05-18

**Authors:** Timothy J. Collier, Nicholas M. Kanaan, Jeffrey H. Kordower

**Affiliations:** 1 Department of Translational Science and Molecular Medicine, Michigan State University, Grand Rapids, Michigan, USA; 2 Mercy Health Hauenstein Neuroscience Center, Grand Rapids, Michigan, USA; 3 Department of Neurological Sciences, Rush University Medical Center, Chicago, Illinois, USA; 4 Center for Neurodegenerative Science, Van Andel Research Institute, Grand Rapids, Michigan, USA

**Keywords:** aging, Parkinson’s disease, nonhuman primates, proteasome, lysosome, oxidative stress, microglia

## Abstract

Despite abundant epidemiological evidence in support of aging as the primary risk factor for PD, biological correlates of a connection have been elusive. In this article, we address the following question: does aging represent biology accurately characterized as pre-PD? We present evidence from our work on midbrain dopamine neurons of aging nonhuman primates that demonstrates that markers of known correlates of dopamine neuron degeneration in PD, including impaired proteasome/lysosome function, oxidative/nitrative damage, and inflammation, all increase with advancing age and are exaggerated in the ventral tier substantia nigra dopamine neurons most vulnerable to degeneration in PD. Our findings support the view that aging-related changes in the dopamine system approach the biological threshold for parkinsonism, actively producing a vulnerable pre-parkinsonian state.

An association between aging and Parkinson’s disease (PD) has been recognized for several decades.^1,2^ Aging is the number one risk factor for the development of PD and is incorporated into some models of its etiology.^3–5^ However, this critical variable is rarely incorporated into preclinical tests of therapeutic interventions for PD,^6,7^ perhaps reflecting skepticism regarding the importance of a biological link between them. The incorporation of aging as a variable in these studies is further complicated by difficulty obtaining aged animals, the high risk of mortality of these subjects, and the exorbitant costs associated with such studies. When considering the association between aging and PD, epidemiological studies support a connection. It is clear that PD prevalence increases with increasing age. A meta-analysis of 47 epidemiological studies of PD worldwide spanning the years 1985 to 2010 confirms the significant rise in the prevalence of PD with advancing age, progressively increasing approximately 10-fold between the ages of 50 and 80.^8^ Also, it is known that the age at onset of PD significantly affects the phenotype and progression of disease,^9–11^ suggesting that biological aging provides distinct starting points for the evolution of PD. Early-onset individuals (age 45–50) typically exhibit a tremor-predominant syndrome, more rapid development of levodopa-induced dyskinesias, and a slower rate of disease progression. Late-onset individuals (age 70 and older) tend to have more severe motor deficits, a postural instability gait disorder, lower susceptibility to levodopa-induced dyskinesias, and more rapid disease progression. Furthermore, evidence from both preclinical and clinical studies of cell transplantation indicate that despite implantation of immature DA neurons that are at a developmental stage optimal for neurite outgrowth and innervation, the environment of the aged brain inhibits functional reconstitution otherwise present in younger individuals.^7,12,13^ There are biological variables that could explain many of these features such as the difference in neuronal progenitor proliferation, integration, innervation, and function between young and aged individuals; however, this concept would need further empirical verification.

Early histological studies that focused on neuron loss in the substantia nigra (SN) left room for the interpretation of the strength and relevance of any connection. Although the majority of studies examining the magnitude of SN cell loss during “normal” aging are in agreement that cell numbers decline at a rate of approximately 7% per decade during the lifespan,^14^ it is clear that at this rate, aging alone cannot account for the cell loss in PD. However, the trajectory of increasing SN cell loss with advancing age is consistent with a pre-PD state as well as the impairments in motor function that are commonly associated with aging (eg, refs. ^15,16^). An enduring argument against an aging–PD connection is based on the anatomical pattern of SN neuron loss, with the greatest magnitude of loss in PD localized to ventral tier SN neurons, and in aging, dorsal tier SN neurons.^17,18^ It is notable that this study used quantification methods that were standard for that time, but would not be acceptable by today’s standards, using manual cell counts from a single 12-μm thick section from the caudal SN. Conclusions drawn from these reports ranged from the contention that PD represents accelerated aging,^19,20^ to the view that aging and PD are distinct and potentially unrelated.^17^ So, the question remains the following: does aging represent biology accurately characterized as pre-PD?

If we accept that SN neuron loss in successful aging cannot account for the substantial loss in PD and that a large portion of the aging population live their lives without developing PD, support for the aging–PD connection may be addressed best at the level of SN neuron biology. If aging and PD have a connection at the level of cellular mechanisms, there should be evidence that important features of this biology are shared and exist along a continuum.

## Evidence From Aging Nonhuman Primates

We revisited the association of aging and PD by incorporating 2 main features into our experimental design. First, we focused our studies on the established regional pattern of ventral midbrain DA neuron degeneration characteristic of PD: profound loss of neurons in the ventral tier of SN (vtSN), less degeneration of dorsal tier SN neurons (dtSN), and relative sparing of adjacent ventral tegmental area neurons (VTA). Second, we moved away from the examination of overt cell loss to study histological markers of the DA neuron phenotype that typically decrease as an early feature of PD^21–23^ and markers of processes known to contribute to degeneration of SN neurons that typically increase during the course of PD. To support a connection between aging and PD, we hypothesized that the magnitude of change in these markers should increase with advancing age and vary in a pattern consistent with regional differences in vulnerability to degeneration of midbrain DA neurons in PD: an aging-related decline in phenotype markers and progressive increase in prodegeneration markers with the greatest magnitude of change in vtSN and less change in the dtSN and VTA.

We used a collection of tissue from rhesus monkeys ranging in age from 9 to 31 years with an estimated aging rate for this species of 3:1 relative to humans (human equivalent of 27–93 years of age).^24^ Of importance to our analysis, nonhuman primates display clear anatomical distinctions between vtSN, dtSN, and VTA similar to humans.

**FIG. 1:**
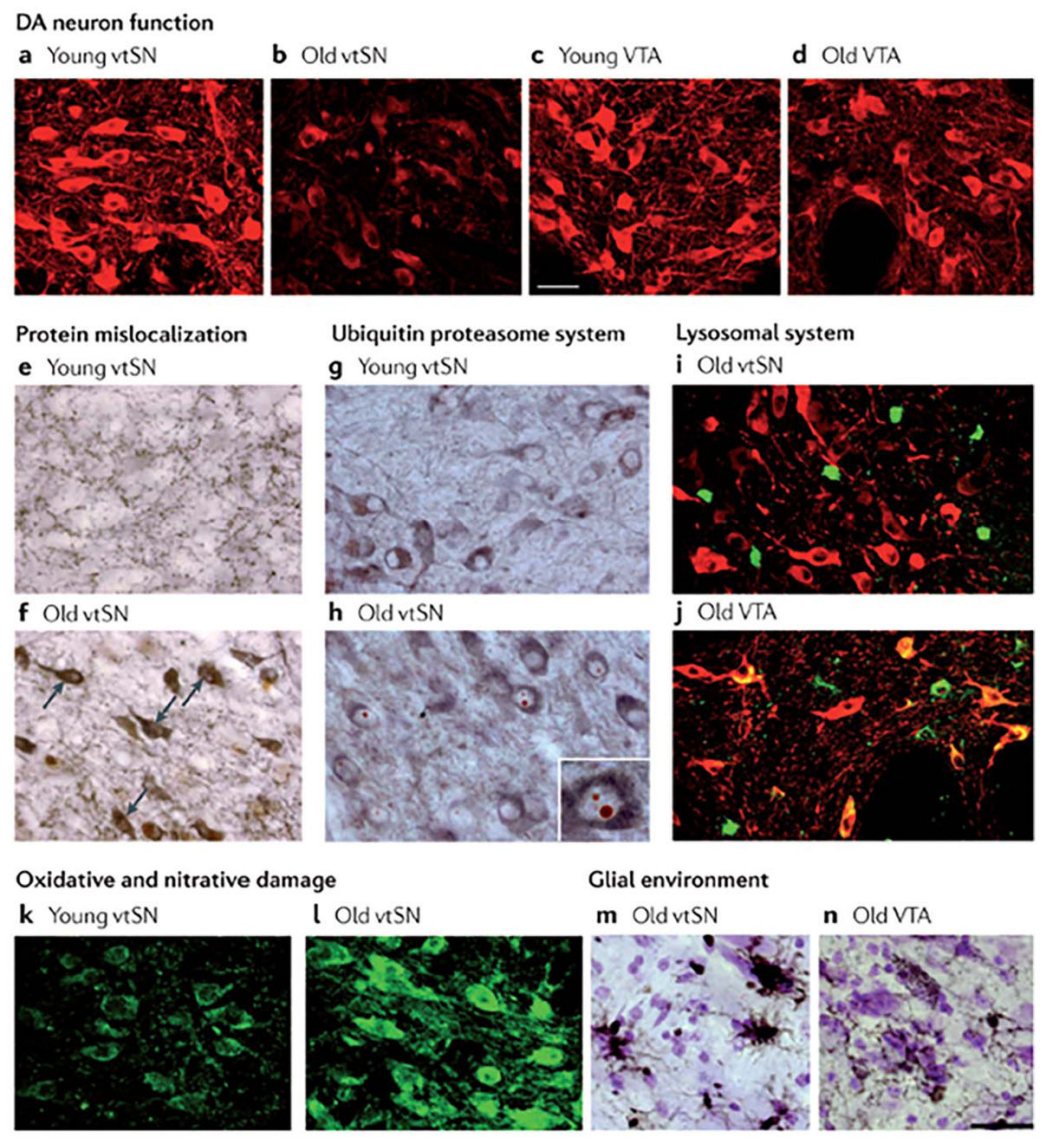
The pattern of ageing-related changes in markers of cellular mechanisms. With advancing age, DA neurons in the ventral tier of the substantia nigra (vtSN)—the population that is most vulnerable to degeneration in PD—show changes with aging. **a–d**: Age-related decline in tyrosine hydroxylase staining (shown in red) in vtSN neurons, but not in vental tegmental area (VTA) neurons. **e,f**: Accumulation of cytoplasmic α-synuclein (shown in brown). Tyrosine hydroxylase staining is shown in gray. Arrows show examples of cytoplasmic α-synuclein in aged vtSN. **g,h**: Increased numbers of Marinesco bodies, characterized by cytoplasmic inclusions of ubiquitin (shown in red). Tyrosine hydroxylase staining is shown in gray. The inset (in part h) is a higher magnification view of a tyrosine hydroxylase immunoreactive neuron of the vtSN exhibiting multiple Marinesco bodies. **i,j**: No accumulation of lipofuscin (shown in green). Tyrosine hydroxylase staining is shown in red, colocalization of lipofuscin and tyrosine hydroxylase is shown in yellow. Note that virtually all lipofuscin staining in the vtSN is not in dopamine neurons, whereas colocalization is apparent in aged VTA neurons. **k,l**: Accumulation of 3-nitrotyrosine (shown in green). **m,n**: Greater microglial reactivity in aged vtSN neurons than in aged VTA neurons, shown by greater staining for human leukocyte antigen (HLA) class II histocompatibility antigen, antigen D related (DR) α-chain (HLA-DRA; a marker for microglia), shown in brown. UPS, ubiquitin–proteasome system. From Ref. ^5^ with permission.

Tyrosine hydroxylase (TH) is the rate-limiting enzyme in synthesis of catecholamine neurotransmitters and is a common histological marker for the DA neuron phenotype. Although large-scale overt loss of DA neurons is not associated with normal aging in nonhuman primates, aging-related declines in staining for TH is readily observed,^25–28^ and the loss of TH phenotype is an early stage of the degenerative process in PD.^22^ Our observations confirmed that no significant overt loss of SN DA neurons occurred in aged nonhuman primates, but an aging-related decline in staining for TH in midbrain DA neurons was present and significantly exaggerated (−63%) in the vtSN neurons most vulnerable to degeneration in PD^29^ (Fig. 1a–d).

Multiple findings implicate impaired cellular mechanisms for clearing abnormal proteins and organelles in the pathobiology of PD, and we have demonstrated that these same factors are found in normal primate aging (Fig. 1e–n). In PD, Lewy bodies diagnostic for PD are ubiquitin-positive cytoplasmic inclusion bodies containing multiple aberrant proteins.^30–32^ The an E3 ubiquitin ligase (PARKIN) genetic form of PD is a mutation of a gene involved in the ubiquitin-proteasome system,^33^ and in concert with PTEN-induced putative kinase 1 (PINK1) mediates elimination of defective mitochondria.^34,35^ In addition, PD postmortem tissue exhibits histological markers of compromised proteasome and lysosome function.^36–39^ Aged nonhuman primates do not develop Lewy bodies, but the efficiency of intracellular clearance mechanisms can be assessed by the presence of ubiquitin-positive inclusions in the nucleus of neurons (Marinesco bodies) and the accumulation of cytoplasmic lipofuscin, a byproduct of lysosome activity associated with the removal of defective organelles including mitochondria.^40^ These markers exhibited increased prevalence with advancing age and were variably expressed in ventral midbrain DA neuron populations.^29^ Ubiquitin-positive nuclear inclusions increased dramatically with advancing age and were nearly exclusively found in the most vulnerable vtSN neurons. Interestingly, the subpopulation of TH+ neurons that contained the ubiquitin inclusions showed a premature reduction of TH staining in middle-aged animals, suggesting a decline in the health or functionality of those neurons. In contrast, cytoplasmic accumulation of lipofuscin followed an opposite pattern, being most prominent in degeneration-resistant VTA neurons and nearly absent in vulnerable vtSN neurons. The presence of lipofuscin also showed a relationship to the TH phenotype. Middle-aged monkey dtSN neurons that contained lipofuscin exhibited an intensity of TH staining comparable to young adult animals, appearing resistant to the normal aging-related decrease in expression of this phenotypic marker. This may represent more efficient removal of damaged or malfunctioning organelles (such as mitochondria) in degeneration-resistant DA neurons. These findings are consistent with the view that aging is associated with a progressive, region-specific, impairment of proteasome function and reduced lysosome function predominantly in the DA neurons most vulnerable to degeneration in PD. Another aspect of age-related neurodegenerative processes is the fact that PD virtually always starts unilaterally and progresses assymetrically. Furthermore, PD occurs more frequently in males. These features deserve further scrutiny from an aging perspective.

Oxidative and nitrative stresses are considered major factors in PD neurodegeneration. Postmortem PD brains exhibit an increase in markers of oxidative damage to proteins,^41^ lipids,^42^ and DNA.^43^ DA neurons are inherently vulnerable to these stressors in part because of the high potential for auto-oxidation of intracellular DA that is not efficiently sequestered into synaptic vesicles.^44,45^ We examined the accumulation and distribution of 3-nitrotyrosine (3NT) in DA neuron populations during aging as a marker of oxidative/nitrative damage to proteins. In addition, we stained for markers of the dopamine transporter responsible for recapturing released DA back into the neuron and the vesicular monoamine transporter type-2 responsible for repackaging intraneuronal DA into vesicles to assess the neuron’s risk for oxidation of free cytoplasmic DA. The 3NT exhibited a progressive increase during aging, and the vulnerable vtSN had significantly more 3NT-positive neurons when compared with the other subregions in all age groups.^46^ Moreover, vtSN neurons with higher 3NT levels showed the strongest expression of transporters and the highest dopamine transporter/vesicular monoamine transporter ratio, suggesting that vulnerable vtSN neurons have the greatest capacity to accumulate free cytosolic DA available for oxidation.

Finally, we examined the intensity, morphology, and distribution of markers for astrocytes and microglia (human leukocyte antigen- antigen D related (HLA-DR)) in ventral midbrain regions during aging.^47^ Activated glial cells are an established correlate of neuro-inflammation, and their pro-inflammatory cytokine products are increased in postmortem PD brains.^48,49^ We detected no differences in the intensity of staining, morphology, or distribution of astrocytes in the ventral midbrain of aging nonhuman primates. In contrast, microglia exhibited increased staining intensity and transformation to activated/phagocytic morphology during aging that preferentially occurred in the PD-vulnerable vtSN region. We extended these findings using MPTP, a DA neurotoxin, to model PD in young-, middle-, and old-aged monkeys. In response to MPTP, the vtSN in the aged monkeys showed an exaggerated microglial response when compared with other subregions, suggesting that the vtSN is more prone to neuroinflammation following an insult.^50^

Our analysis of markers of multiple factors implicated in the complex etiology of DA neuron degeneration in PD, and their preferential expression in DA neuron populations that are selectively vulnerable to degeneration in the disease leads to the conclusion that aging itself is associated with changes consistent with a pre-PD state. During normal aging in nonhuman primates, the DA neurons most vulnerable to degeneration in PD display histological correlates of progressive loss of phenotype, the accumulation of ubiquitin-positive inclusions, inadequate lysosome function, increased oxidative/nitrative damage, and exaggerated neuroinflammation, all factors implicated in PD pathology. Aging and PD exhibit region-specific shared biology for these factors, suggesting that they exist along a shared continuum.

## Additional Considerations

### Alpha-Synuclein (α-syn)

**FIG. 2:**
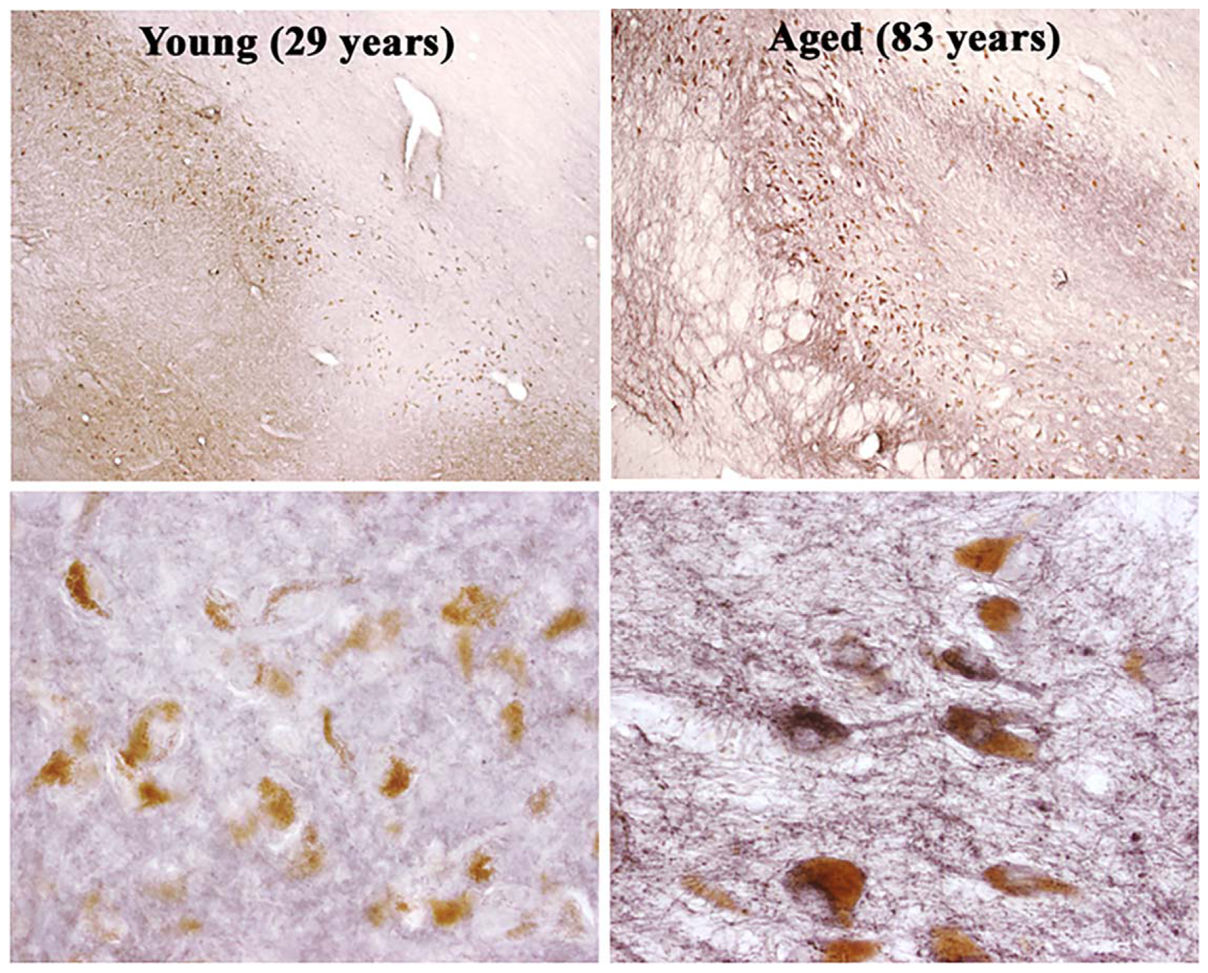
Age-related accumulation of alpha synuclein within the human substantia nigra.

Misfolded α-syn is a major component of the Lewy body inclusions that characterize PD pathology,^30^ and genetic forms of the disease are associated with the mutation and multiplication of the α-syn gene.^51–54^ Although Lewy bodies are not a common feature of normal aging, α-syn does display a change in intra-neuronal localization during aging, and this change is virtually identical in both humans and nonhuman primates.^55^ In younger individuals, α-syn is enriched in synaptic terminals and below the level of detection in the soma of neurons. With aging, there is a prominent increase in staining for α-syn within the cytoplasm of SN neuron cell bodies (Fig. 2). This increase in somatic α-syn is correlated with reductions in DA phenotype markers of SN neurons.^55^ Pathological conformations of α-syn present in Lewy bodies are believed to originate from increased levels of the soluble forms of the protein.^56^ In fact, the pathological increase in the monomeric and/or tetrameric soluble forms of α-syn seen in aging and PD is a proposed therapeutic target for PD, and the aging-related accumulation of α-syn in the soma of neurons likely represents a pre-Lewy body state. Consistent with this, Markesbery and colleagues^57^ detected α-syn-positive SN Lewy body pathology in 20% of 139 autopsy cases of elderly individuals (69–101 years) free of movement disorders. These authors draw the conclusion that this aging-related pathology likely represents presymptomatic PD.

### Evidence From *Caenorhabditis elegans (C. elegans)*

The worm *C. elegans* provides a powerful experimental approach for studying the genetics of aging and PD. A recent report by Cooper and colleagues^58^ examined the effects of delaying aging on the phenotype of worms harboring human PD mutations in α-syn or leucine-rich repeat kinase 2 (LRRK2), specifically in DA neurons. PD-mutant worms exhibited normal development and lifespan, but expressed a phenotype that included deficits in dopamine-dependent behaviors, greater sensitivity to stressors, and accelerated DA neuron degeneration. PD-mutant worms were crossed with long-lived worms expressing a mutation in the gene encoding insulin-like growth factor 1 (*daf-2*) that decreases insulin/insulin-like growth factor 1 (IGF-1) signaling.^59^ PD-mutant/*daf-2* mutant worms showed a doubling of lifespan, indicating that the genetic cross achieved the desired anti-aging effects. A comparison of the phenotypes of PD-mutant worms and PD/*daf-2* mutant worms at the same stage of adulthood indicated that delaying aging reduced or prevented all PD-related features, increasing the survival of DA neurons, ameliorating deficits in DA-dependent behaviors and a reversal of the increased sensitivity to stressors.

### Evidence From Human Induced Pluripotent Stem Cells (iPSC)

IPSCs are produced by in vitro reprogramming of somatic cells, often skin fibroblasts, to revert to an embryonic, pluripotent state.^60^ One goal of iPSC research is the potential to use these cells as in vitro models of diseases influenced by genetic risk factors, including PD. For PD, multiple lines of these cells have been derived from patients carrying genetic predispositions including homozygous mutations in PINK1 or PARKIN. When these cells are further reprogrammed to differentiate into midbrain DA neurons, they exhibit mild phenotypic changes with virtually no overt degenerative features.^61–63^ However, as expected, the generation of iPSCs resets the biology of these cells to an immature, stem cell state. In a recent report, Miller and colleagues^64^ developed the argument that this rejuvenation associated with creation of iPSCs may contribute importantly to the absence of a degenerative phenotype in PD iPSC-derived DA neurons by removing aging as a factor in modeling a late-onset disease. These investigators went on to demonstrate that cell markers of aging can be induced in iPSCs by increasing the expression of the protein progerin, a splice variant of the nuclear envelope protein Lamin A. The accumulation of progerin is a feature of aged cells and is associated with the parallel increased expression of multiple markers of cell aging. Following an induction of in vitro aging via increased expression of progerin, the unremarkable phenotypes of PD iPSC-derived DA neurons shift to exhibit properties that are typical of degenerating DA neurons in PD, including increased apoptosis, dendrite shortening, and reduced activation of Akt signaling pathways. These investigators went on to transplant PD iPSC-DA neurons/progerin+ cells into a parkinsonian rat model. Again, these grafted neurons exhibited several properties consistent with DA neurodegeneration: reduced capacity to ameliorate behavioral deficits, progressive loss of TH+ phenotype, neurite degeneration, folded nuclear morphologies, accumulation of neuro-melanin, enlarged mitochondria, and the presence of multilamellar inclusions postulated to be precursors of Lewy bodies. The authors concluded that these findings supported the presence of a synergistic interaction between PD genotypes and aging.

### Genetics of Central Nervous System Aging

Work by Glorioso and colleagues^65^ identified a transcriptome signature of normal human brain aging based on the microarray analysis of 4 regions (2 regions of prefrontal cortex, anterior cingulate cortex, and amygdala) in 2 cohorts of individuals aged 14–79 years collectively (N = 75), free of neurological disease by clinical assessment and postmortem pathology. A set of 356 age-regulated genes that exhibited changes conserved across brain regions was found to reliably predict the chronological age of an individual. The analysis of a set of 3935 genes, not restricted by significance in all brain regions, indicated that neurological disease-related genes represented 34% of all age-regulated genes. In contrast, disease-related genes represented only 4% of genes not regulated by aging. A total of 170 PD-related genes were among those identified as age regulated. The analysis of a subset of genes with strong associations to neurological disease, including PD genes Parkin, ubiquitin carboxy-terminal hydrolase L1 (UCHL1), Pink-1, and protein deglycase DJ-1 (DJ-1), uniformly exhibited aging-related changes in expression in a direction consistent with promotion of disease (32 of 33 genes studied). The authors offered the conclusion that the overrepresentation of genes related to neurological diseases among genes that are aging regulated and the consistent directionality of change toward the promotion of disease supports the view that age-gated diseases, including PD, may represent exaggeration of changes in gene expression intrinsic to normal aging.

## Conclusions

**FIG. 3:**
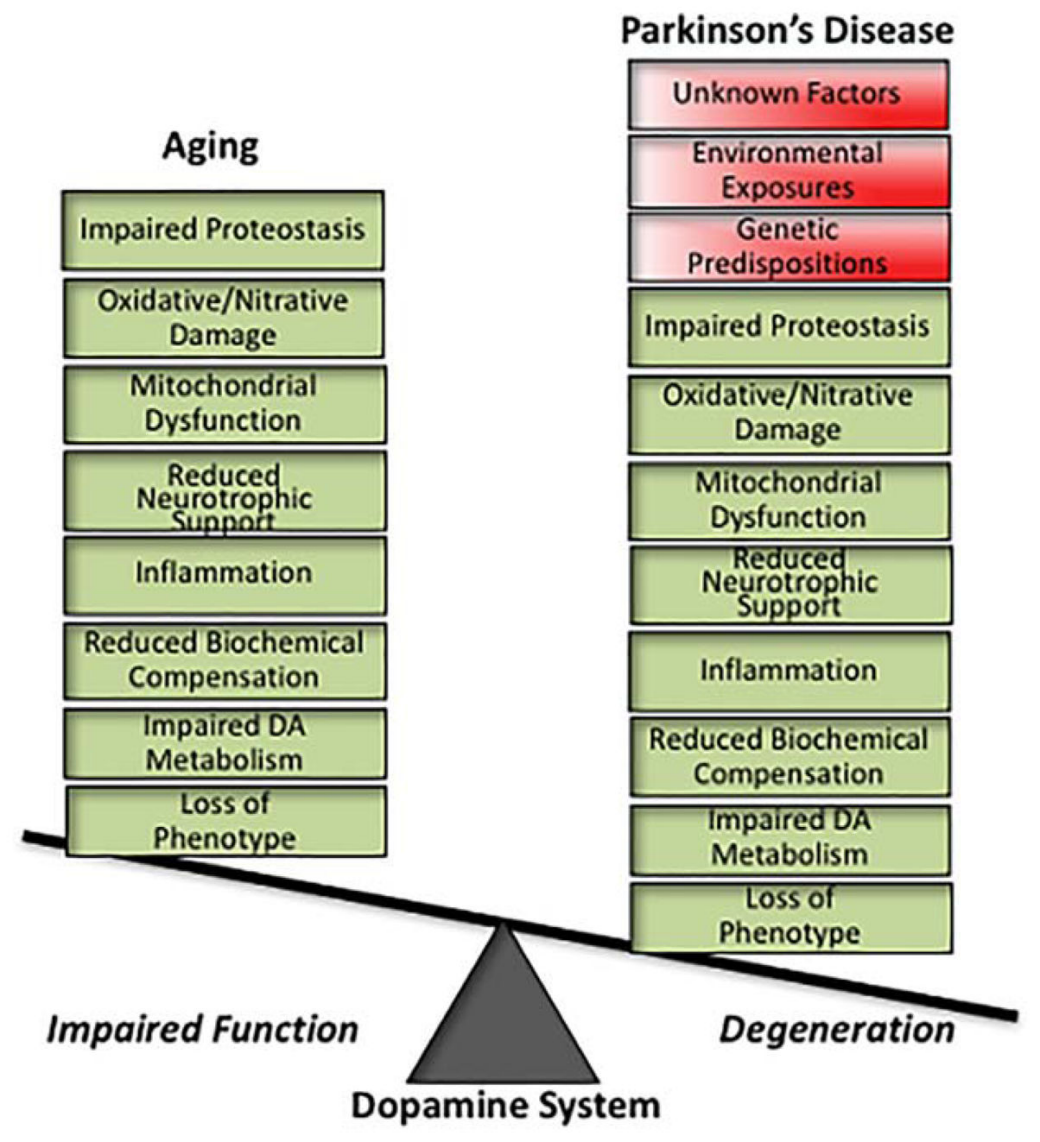
The nigrostriatal dopamine system in aging and PD share important biological features. DA neurodegeneration in PD presents a complex biology of interacting factors. Many of these factors also are present during aging of this system, exhibiting the same direction of change while varying in the magnitude of change. In normal aging, threats to dopamine neuron viability are expressed as impaired function of the system, whereas additional contributions of genetic, environmental, and other unknown factors exaggerate aging-related changes to reach the threshold for dopamine neurodegeneration and symptomatic PD. The multiple shared features of dopamine neuron biology in aging and PD lead to the hypotheses that aging actively creates a pre-PD state and that aging-related changes are the pathological foundation on which the degeneration in PD is built.

Returning to the original question of whether aging is pre-PD, the short answer is “yes.” At a simplistic level, there is no PD without aging (although one does not need to be elderly for PD to occur). Even dominantly inherited genetic forms of PD require the passage of time, often decades, for PD symptoms to emerge. Without aging, degenerative changes in DA neurons are not fully expressed in PD genetic models in *C. elegans*^58^ and human iPSCs,^64^ and the expression of genes strongly associated with PD are uniformly regulated by aging in a disease-promoting direction.^65^ On the flip side, aging is not PD. Even in individuals aged 80 years and older, the prevalence of PD is in the range of 1% to 2% of the population.^8^ However, at least for the nigrostriatal DA system, our evidence from nonhuman primates supports the view that aging and PD share important biological features (Fig. 3). Our previous studies demonstrated that aging is associated with declines in spontaneous motor activity, striatal DA levels, TH phenotype of SN neurons, and failure to engage a compensatory increase in DA system function in response to a toxic insult that is a normal response in younger adult monkeys.^25^ In addition, we have shown that the aged striatum represents an impoverished trophic environment for the axon projections of SN DA neurons.^7,66,67^ Our more recent work demonstrates that markers of known correlates of DA neuron degeneration in PD, including impaired proteasome/lysosome function, oxidative/nitrative damage, and inflammation, increase with advancing age and are exaggerated in the ventral tier SN DA neurons most vulnerable to degeneration in PD. This shared biology and directionality of changes suggests that aging actively creates a vulnerable preparkin-sonian state, which has important implications for aging as a critical variable in PD modeling and the vetting of potential therapeutic interventions.
